# Heterotrophic N_2_-fixation contributes to nitrogen economy of a common wetland sedge, *Schoenoplectus californicus*

**DOI:** 10.1371/journal.pone.0195570

**Published:** 2018-04-23

**Authors:** Eliška Rejmánková, Dagmara Sirová, Stephanie T. Castle, Jiří Bárta, Heather Carpenter

**Affiliations:** 1 Department of Environmental Science and Policy, University of California, Davis, United States of America; 2 Institute of Hydrobiology, Biology Centre CAS, České Budějovice, Czech Republic; 3 University of South Bohemia, České Budějovice, Czech Republic; Universite de Sherbrooke, CANADA

## Abstract

A survey of the ecological variability within 52 populations of *Schoenoplectus californicus* (C.A. Mey.) Soják across its distributional range revealed that it is commonly found in nitrogen (N) limited areas, but rarely in phosphorus limited soils. We explored the hypothesis that *S*. *californicus* supplements its nitrogen demand by bacterial N_2_-fixation processes associated with its roots and rhizomes. We estimated N_2_-fixation of diazotrophs associated with plant rhizomes and roots from several locations throughout the species’ range and conducted an experiment growing plants in zero, low, and high N additions. Nitrogenase activity in rhizomes and roots was measured using the acetylene reduction assay. The presence of diazotrophs was verified by the detection of the *nifH* gene. Nitrogenase activity was restricted to rhizomes and roots and it was two orders of magnitude higher in the latter plant organs (81 and 2032 nmol C_2_H_4_ g DW^-1^ d^-1^, respectively). Correspondingly, 40x more *nifH* gene copies were found on roots compared to rhizomes. The proportion of the *nifH* gene copies in total bacterial DNA was positively correlated with the nitrogenase activity. In the experiment, the contribution of fixed N to the plant N content ranged from 13.8% to 32.5% among clones from different locations. These are relatively high values for a non-cultivated plant and justify future research on the link between N-fixing bacteria and *S*. *californicus* production.

## Introduction

Growth of terrestrial as well as wetland plants in non-agricultural settings is often limited by the availability of nutrients, specifically nitrogen (N) and phosphorus (P) [1, 2]. Plants have evolved two broad strategies to deal with nutrient-limiting environments: (1) conservation of use; and (2) enhanced acquisition [3, 4, 5]. Resorption of nutrients from senescing to newly growing or storage organs is a typical example of a conservation of use strategy. The enhanced acquisition involves production and secretion of hydrolytical enzymes such as phosphatases in case of P limitation, or the utilization of rhizosphere bacteria that can increase the bioavailability of N through N_2_-fixation [6]. Bacteria capable of performing N_2_-fixation can colonize both root surfaces (and are referred to as “epiphytes” here) as well as the internal tissues (“endophytes”) (for review see [7]. The reduction of N_2_ to ammonia during its biological fixation is an energetically expensive process and the input of easily available C from roots into the plant rhizosphere can sustain high activity of root associated diazotrophs [8, 9, 10, 11]. Epiphytic diazotrophs establishing loosely organized associative relationships in the rhizosphere have been documented frequently for tropical grasses [8, 12, 13] and among wetland plants for littoral macrophytes [14, 15, 16]. Endophytic relationships of diazotrophs have been reported in a variety of plant roots and rhizomes including sugar cane, *Sorghum*, *Miscanthus*, and others [17, 18, 19, 20, 21, 22]. Here we will focus on the potential role of both epiphytic and endophytic nitrogen fixation in the nutrient economy of a giant bulrush, *Schoenoplectus californicus*.

**Fig 1:**
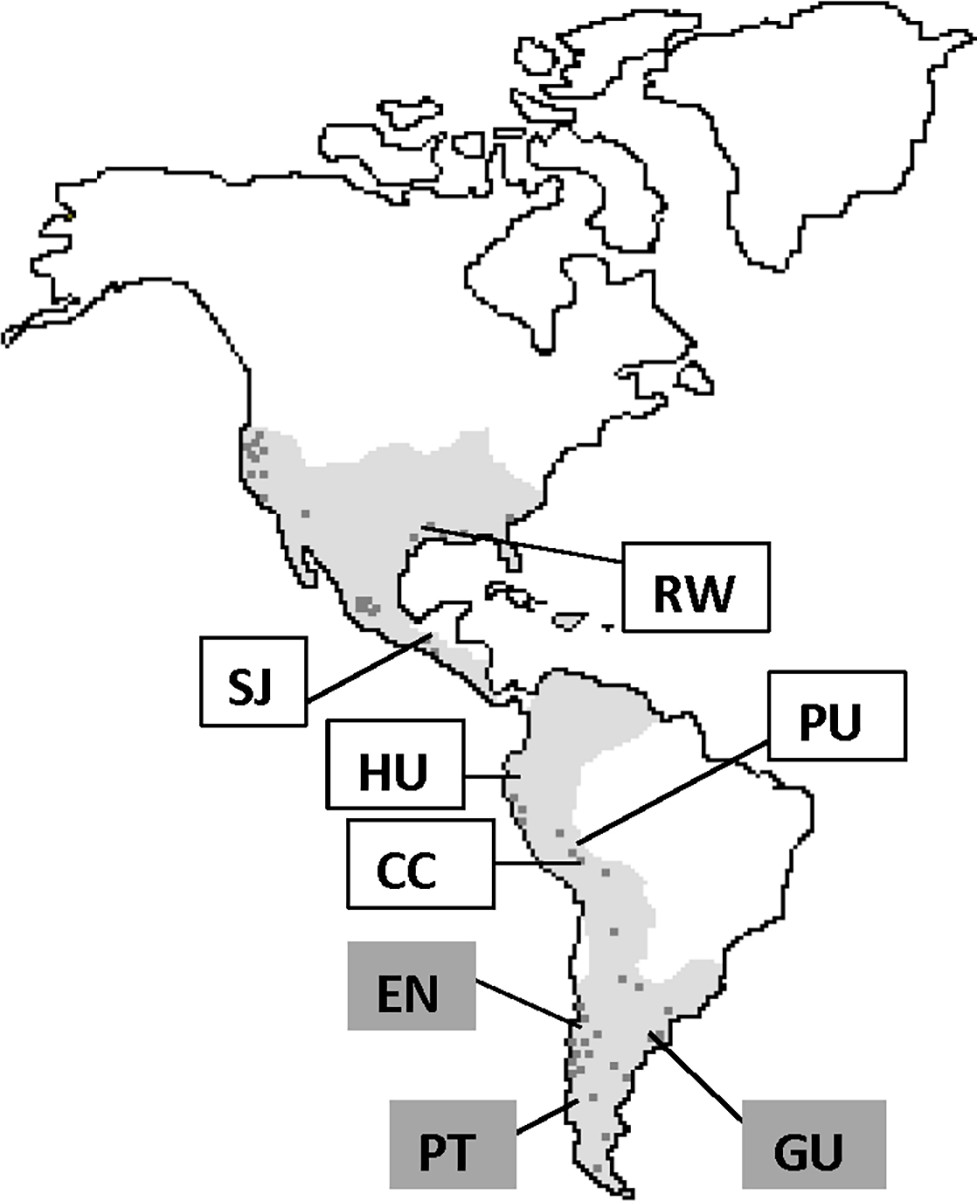
Geographic origins of the clones used in the cultivation experiment. The distribution of *Schoenoplectus californicus* in the Americas is shown in grey, the dots indicate the sampling locations [23]. The clones included in this study are indicated by letters: Variety *tereticulmis* EN: Ensenada, Chile; GU: Guillermo, Argentina; PT: Puente, Chile. Variety *californicus* CC: Copacabana, Bolivia; PU: Puno, Perú; HU: Huanchaco, Perú; SJ: San Juan, Guatemala; RW: Rockefeller, Louisiana.

*Schoenoplectus californicus* (Cyperaceae) is a large, perennial, rhizomatous wetland sedge reaching up to 6 meters in height and often forming monospecific stands [23]. Two varieties with similar ecology, var. *californicus* (C. A. Meyer) Soják and var. *tereticulmis* (Steud.) Vegetti, are present in the southern part of its distributional range. As a dominant producer of biomass, *S*. *californicus* can impact biogeochemical cycles by providing a source of organic material and by oxygenating the rhizosphere [24]. In many regions, it plays an important role in the human economy providing raw materials for the construction of boats, all-purpose mats and handicrafts [25, 26]. In the survey of the ecological variability within 52 populations of *S*. *californicus* throughout the Western Hemisphere (Fig 1) we noticed that availability of phosphorus (P) appears to be important and this species is rarely found in P limited soils [23]. While common in the P-rich soils of Chile and the Central American highlands, the species is absent from the predominantly P limited ecosystems of the Yucatan peninsula and Cuba. Contrastingly, it is commonly found in areas known to be N limited, such as the Orinoco delta of Venezuela, the delta of the Paraná River in Argentina, and the Central Valley of California. In the entire data set from Carpenter [23], the average total soil P was 0.74 mg g^-1^, which is well over 0.5 mg g^-1^ regarded as a sufficient amount of P for wetland sediments [27]. In contrast, N availability was found to be variable but generally on the low side (average total soil N of 5.5 mg g^-1^), with about half of the locations containing < 4 mg g^-1^. No differences were found in biomass production among the populations from sites with N-limited sediments, nor did there seem to be less N in plant tissue (Table 1), therefore positive association with N-fixing diazotrophs was suspected.

**Table 1:** Soil and plant total nitrogen, TN, and phosphorus, TP, mg g^-1^, and aboveground biomass, W, from 52 populations of *Schoenoplectus californicus* [23].

		SOIL			PLANT TISSUE			PLANT BIOMASS
	n	TN	TP	N/P	TN	TP	N/P	W, g m^-1^
Average all	52	5.5	0.75	7.2	16.9	1.37	13	1325
Low N group	22	**1.75**	0.50	4.1	**16.6**	1.50	11.7	**1364**
High N group	30	**8.40**	0.91	9.5	**17.1**	1.30	14.0	**1286**
*P value (Mann-Whitney U-test)*		***0*.*001***	*0*.*01*	*0*.*001*	***NS***	*0*.*05*	*0*.*05*	***NS***

Data presented as means for the whole data set and means for low vs. high nitrogen. Note: Soil TN < 4 mg g^-1^ and TP < 0.5 mg g^-1^ in wetland sediments indicate potential N or P limitation [28]; the average tissue N in other Cyperaceae species from the N-limited region was 13.1 mg g^-1^ (n = 10; SD = 0.24). Biomass sampled in 2007 in the middle of the growing period (Dec-Feb Southern hemisphere, June-August Northern hemisphere).

Biological nitrogen fixation, BNF, belongs to the most essential biological processes and its knowledge is critical to our understanding of biogeochemical ecosystem functioning [28, 29, 30]. It occurs via two primary pathways: symbiotic and nonsymbiotic, and is performed by prokaryotic organisms called diazotrophs that can be either autrotrophic (cyanobacteria), or heterotrophic (numerous genera of bacteria such as *Azospirillum*, *Herbaspirillum* and others [31]). Symbiotic N_2_-fixation is defined as the biological reduction of N_2_ occurring through mutualistic relationships between microorganisms (e.g., rhizobia) and plant roots (e.g., legumes). Nonsymbiotic nitrogen fixation includes fixation by the true free-living diazotrophs (e.g.,heterotrophic N_2_-fixation in leaf litter and soil or water) [31, 32], as well as by autotrophic and heterotrophic organisms of intercellular and epiphytic growth associated with certain species of bryophytes [33]. In the past, the majority of studies dealt with the symbiotic N_2_-fixation in legumes, however more recently, the important role of free-living and epiphytic N_2_-fixation is being acknowledged and current evidence suggests that free-living N_2_-fixation represents a critical N input to many terrestrial and aquatic ecosystems, particularly those lacking large numbers of symbiotic N_2_-fixing plants [11, 21]. Most of the studies on endophytic N_2_-fixation have been focused on crop plants such as sugar cane and rice, while only a few studies on natural plant populations are available and none, to our best knowledge, reports endophytic diazotrophs from any Cyperaceae family. Field experiments have demonstrated that 60% or more of plant N may be provided by plant-associated biological N_2_-fixation in sugarcane [34, 35].

With the rapid development of molecular methods, many laboratories are now reporting on the actual bacterial composition of both epiphytic and endophytic diazotrophs, or at least the detection of *nifH* gene encoding the Fe protein polypeptide of the nitrogenase enzyme. The detection of the *nifH* gene in the genomic DNA sample labels that particular isolate as a potential diazotroph [36]. Expression of genes associated with N_2_ fixation (*nifH*) has been demonstrated multiple times [37, 38].

Here we report the N_2_-fixation activities associated with roots and rhizomes of a common macrophyte, *Schoenplectus californicus*. Our goal was to answer the following questions related to both the basic understanding of *S*. *californicus* associated N_2_-fixation processes as well as applied aspects aimed at potential economical utilization of this plant:

- Are there any differences between nitrogenase activity of roots (epiphytic) and rhizomes (endophytic) associated diazotrophs and what proportion of plant N budget has been derived from N_2_-fixation? We predicted that root associated N_2_-fixation will be higher due to the higher population densities of rhizobacteria as compared to endophytic bacteria.
- Does N_2_-fixation differ in *S*. *californicus* populations from nutrient enriched (polluted) *vs*. oligotrophic (unpolluted) locations? We predicted that N_2_-fixation will be higher in the unpolluted areas.
- Are there any differences in N_2_-fixation between the two varieties, *S*. *californicus* var. *tereticulmis* and var. *californicus*?
- Are the presence of the *nifH* gene and/or the δ^15^N signature of shoots good predictors of N_2_-fixation?

## Material and methods

The study has three components:

1. Preliminary survey of epi- and endophytic N_2_-fixation associated with *S*. *californicus* populations from different parts of its wide range of distribution.
2. Regional study of epi- and endophytic N_2_-fixation associated with *S*. *californicus* populations from the littoral zones of Lake Atitlán, Guatemala, assessing the variability of the two processes at nutrient poor and nutrient rich sites.
3. Controlled growth experiment with two varieties of *S*. *californicus*, originating from eight different locations throughout its distribution range, at three different N levels with a labeled ^15^N source to determine the contribution of N_2_-fixation by epi- and endophytic diazotrophs to the plant’s growth.

The field permit to do research at Lake Atitlán was issued by the Autoridad para el Manejo Sustenable de la Cuenca del Lago Atitlán y su Entorno (AMSCLAE); no other field permits were required.

Nitrogenase activity, delta ^15^N signature of shoots and/or rhizomes, and the presence of the *nif*H gene (see below) were used as indicators of a fixing capability of diazotrophs associated with the respective *S*. *californicus* populations.

### Descriptions of study sites

Table 2 summarizes information on all the locations where the study plants originated from. It includes locations from Carpenter’s [23] survey of the ecological variability within populations of *S*. *californicus* throughout the Western Hemisphere (United States, México, Guatemala, Perú, Bolivia, Argentina, Chile, and Easter Island) done in 2006 and 2007. Rhizomes of clones from the genetically distinct populations were collected and planted at UC Davis where they have been propagated and maintained in outdoor cultivations. Table 2 also includes description of study sites around Lake Atitlán in Guatemala and few additional sites from Central California and Belize.

**Table 2:** Characteristics of sampling locations for *Schoenoplectus californicus*; *tere* = variety *tereticulmis*; *cali* = variety *californicus*.

								Soil nutrients (mg g^-1^ dry soil)				
Location	Abbr.	Species	Variety	Latitude D.d	Longitude D.d.	Altitude m	Temperature ^o^C	Total N	Total P	Soil N/P	Plant N/P	δ^15^N ‰
**PRELIMINARY SURVEY**												
Pucon, Chile *		*S*. *californicus*	*tere*	-39.277	-71.981	214	16.1/7.4	0.6	0.5	1.2	11.7	na
Saramiento, Argentina *		*S*. *californicus*	*tere*	-45.700	-69.162	260	18.9/6.6	1.6	0.5	3.2	11.9	na
Rio Vista, Calif., US *		*S*. *californicus*	*cali*	38.133	-121.68	1	22.2/7.5	0.4	0.3	1.3	14.5	na
Lindsey Slough, Calif., US *		*S*. *californicus*	*cali*	*38*.*262*	-121.79	2	23.9/7.3	1.5	0.7	2.1	9.6	na
Cosumnes, Calif., US		*S*. *acutus*		38.262	-121.438	50	24.0/10.6	1.5	0.6	2.5	7.0	5.4
Cosumnes, Calif., US		*T*. *domingensis*		38.266	-121.439	50	24.0/10.7	1.6	0.6	2.7	9.2	4.7
Deep marsh, Belize		*T*. *domingensis*		18.004	-88.448	5	31.5/20.4	6.2	0.2	31.0	27.5	-0.6
**CASE STUDY AT ATITLÁN**												
Isla de Silencio, Guate.	ISLA	*S*. *californicus*	*cali*	14.666	-91.214	1575	23.1/14.7	0.3	0.3	1.0	11.3	0.9
San Marcos, Guatemala	SM	*S*. *californicus*	*cali*	14.722	-91.251	1575	23.1/14.7	1.7	0.7	2.4	12.5	2.0
San Pedro, Guatemala	SP	*S*. *californicus*	*cali*	14.677	-91.138	1575	23.1/14.7	1.9	0.8	2.4	14.2	-8.2
San Lucas, Guatemala	SL	*S*. *californicus*	*cali*	14.634	-91.138	1575	23.1/14.7	4.3	0.8	5.4	10.6	-3.0
Santiago, Guatemala	SAN	*S*. *californicus*	*cali*	14.635	-91.234	1575	23.1/14.7	4.1	0.9	4.6	13.1	-5.1
Panajab, Guatemala	PAN	*S*. *californicus*	*cali*	14.657	-91.223	1575	23.1/14.7	4.0	0.9	4.4	12.9	0.7
**EXPERIMENT**												
Guillermo, Argentina *	GU	*S*. *californicus*	*tere*	-41.36	-71.515	856	14.2/2.4	3.4	0.5	6.8	6.7	0.3
Puente, Chile *	PT	*S*. *californicus*	*tere*	-41.23	-72.623	9	14.6/6.8	1.3	0.3	4.3	11.9	-0.1
Copacabana, Bolivia *	CC	*S*. *californicus*	*cali*	-16.147	-69.075	3810	12.1/6.9	4.1	0.8	5.2	10.9	1.3
Rockefeller, Louisiana *	RW	*S*. *californicus*	*cali*	29.709	-92.826	10	27.8/10.2	3.9	0.6	8.8	7.1	-4.0
San Juan, Guatemala *	SJ	*S*. *californicus*	*cali*	14.698	-91.284	1575	23.1/14.7	1.9	0.7	2.5	10.3	-0.6
Huanchaco, Peru *	HU	*S*. *californicus*	*cali*	-8.068	-79.123	3	13.9/10.0	8.5	1.2	7.1	14.7	22.6
Ensenada, Chile *	EN	*S*. *californicus*	*tere*	-41.653	-73.562	62	14.6/6.8	7.4	0.9	8.5	15.2	7.8
Puno, Peru *	PU	*S*. *californicus*	*cali*	-14.148	-69.689	3810	12.1/6.9	7.0	1.1	6.4	28.5	5.7

The sites of Carpenter’s survey from 2007 [23] are marked with *. Clones from all these locations have been since in the common garden cultivation at UC Davis. The first three locations from Atitlán region are from the unpolluted, the remaining three from polluted locations. The first five locations of the clones used in the experiment are from low nitrogen, the remaining from high nitrogen sampling sites. D.d. = decimal degrees; temperature average high/average low; δ^15^N is from the shoots in the time of collection at original locations.

#### Component 1: Preliminary surveys

Tests of N_2_-fixation capability of epiphytic and endophytic diazotrophs were conducted on *Schoenoplectus californicus* originating in four Central and South American locations and cultivated in the common garden in Davis. To check if the endophytic diazotrophs were also present in rhizomes and shoots of other species of a similar ecological niche, samples of *Schoenoplectus acutus* and a common wetland macrophyte, *Typha domingensis*, from few locations in the Central Valley of California (N-limited) and Belize (P-limited) were surveyed [27] (Table 2). ARA assays were run in hours following sample collection.

#### Component 2: Case study of *Schoenoplectus californicus* populations at Lake Atitlán

To assess the spatial and temporal variability of *S*. *californicus* associated N_2_-fixation and the potential impact of nutrient enrichment, we collected samples from spatially separated populations in the littoral zone of an oligo-to mesotrophic Lake Atitlán, Guatemala in August 2010 and February 2011 (Table 2)—one of the N-limited regions included in the initial Carpenter’s study [23]. Three of the locations were in the unpolluted parts of the lake, while the remaining three were at the proximity to the runoff from human settlements or other nutrient rich sources. The polluted locations were characterized by about five- and two-fold higher concentrations of total N and total P in water, respectively, compared to the unpolluted locations (polluted: TN 525 μg L^-1^, TP 93 μg L^-1^; unpolluted: TN 116 μg L^-1^, TP 50 μg L ^-1^). The polluted locations had also higher TN and TP content in sediments (Table 2).

#### Component 3: Cultivation experiment

From the *S*. *californicus* clone collection, we selected five clones originating in the locations with low N and three in locations with high N (Table 2; Note: Soil TN < 4 mg g^-1^ and TP < 0.5 mg g^-1^ in wetland sediments indicate potential N or P limitation [27]). The selection encompassed the genetic (variety *tereticulmis* and *californicus*) and geographic ranges of the species (Fig 1, Table 2). From now on the two varieties, *tereticulmis* and *californicus*, will be abbreviated as “*tere*” and “*cali*”. The plants were propagated and transplanted for 5 weeks into sterilized sand and tap water (NO_3_-N = 3 ppm; PO_4_-P = 0.2 ppm). At the beginning of the experiment, individual plants comprised of a 7-12cm long shoot with a short piece of rhizome were planted in 3 replicates for each treatment in 3L pots filled with sterilized sand. Since the plants have been in the common garden cultivation for several years, there were no differences among the plants from low *vs* high group in either δ^15^N or % N content (δ15N 4.4 ‰ +/- 0.99 SD and 4.8 ‰ +/-1.04 SD; N content 1.4% +/-0.30 SD and 1.5% +/-0.27 SD for low and high respectively). All pots received 0.25% Hoagland nutrient solution minus N. Hoagland solution was changed three times during the duration of the experiment. The zero, low, and high N treatment received biweekly 0, 20, and 200 mg/pot of N, resulting in the total addition of 0, 120, and 1200 mg N/pot (corresponding to 0, 4, and 40 g N m^-2^). Nitrogen was applied as KNO_3_ with a value δ^15^N of 67.74‰. All pots were placed under ambient environmental conditions in Davis, CA, in a large tub filled with water to prevent overheating. The experiment lasted 87 days (June 3-August 29; during this time, there is typically no rainfall in the Central Valley of California, thus there were no concerns regarding potential uncontrolled N addition by rain). At the end of the experiment, the cumulative length of shoots was recorded, shoots, rhizomes, and roots were separated, washed in DI, and the aliquots were used for measurement of nitrogenase activity. The remaining samples were freeze-dried, weighed, and ground for subsequent determination of δ^15^N, total N, and molecular analyses for *nif*H gene presence and abundance among samples.

### Collection of samples for nitrogenase activity measurements

Four plants at each location (components 1 and 2) and one plant per pot (component 3) were extracted carefully from sediments to prevent extensive root damage and roots and rhizomes were rinsed in surface water to remove adhered larger particles and sealed in a plastic bag. The samples were transported to the laboratory, where the live roots were identified by color and structure, rinsed in distilled water, and an equivalent of ~ 20–40 mg DW was transferred to 40 ml glass test tubes with three replicates per plant. A preliminary test was conducted to verify that this sample manipulation did not impact nitrogenase activity. In the test we compared ARA in root samples treated as described above with those that were collected, quickly rinsed in deoxygenated water and immediately placed to the fixation vials where the oxygen was lowered by exchange of part of the headspace for N_2_ gas. There were no significant differences in the nitrogenase activity measured by ARA for the two treatments over 2, 5, 10, 18 and 25 hours of incubation (t-test, P = 0.7). Rhizome and stem tissues were surface sterilized sequentially in sterile distilled water, 95% ethanol, and 1.6% hypochlorite for 30s each; between each step the tissues were rinsed three times with sterile water [39]. The outer, coarse surface layer of rhizomes was removed after surface sterilization, leaving only tissue that did not come in contact with the soil. Because of the complexity of the root structure, it is difficult to reliably surface sterilize these organs; we decided to consider the root-associated nitrogenase activity as a result of joint activity of both the endo- and epiphytic microorganisms. The tissue was transferred to 40 ml glass test tubes (each in three replicates) with 200 μL (for rhizomes) and 20 mL (for roots) of distilled water and fitted with Teflon septum lids.

### Nitrogenase activity

The acetylene reduction technique, ARA [40], was employed to estimate N_2_-fixation by the reduction of acetylene to ethylene by nitrogenase. Ten percent of the headspace were replaced with acetylene gas, freshly generated from calcium carbide, and the bottles were incubated for 24 hours at 28 degrees C. At the end of the exposure, 7–8 mL of headspace was withdrawn with an airtight syringe (Alltech) and analyzed by gas chromatograph (Shimadzu 14 GC) with a flame ionization detector and a Porapak-T column at 80°C. The results are reported as the nitrogenase activity in nmol C_2_H_4_ g^-1^ d^-1^ of dry weight. Controls run as samples without acetylene addition as well as blanks (tubes without plant tissue incubated with acetylene) showed no endogenous ethylene production. Samples were kept for dry weight determination after terminating the exposure (see [11] for more detailed description).

### Calibration of ARA through ^15^N_2_ reduction assay

On a subset of 13 root and 7 rhizome samples from the experiment, the nitrogenase activity, measured using the ARA, was calibrated by ^15^N_2_ reduction assay. The measurements were conducted at the same time as ARA but 2 mL of ^15^N_2_ (99atom%, Cambridge Isotope) was added instead of C_2_H_2_. At the end of the incubation, the content of the containers was frozen, freeze dried and then ground in a Wiley mill. The initial ^15^N natural abundance of the sample was determined from the ARA samples. The delta^15^N (in relation to atmospheric N_2_ as the reference standard material) was measured by an isotope ratio mass spectrometer (see below). Biomass specific N_2_ fixation rate, normalized to organic N, was calculated as isotopic balance [41, 42]:

$$V(t^{-1})=\left[\frac{\left({AP}_{PN\;final}-{AP}_{PN\;initial}\right)}{\left({AP}_{N2}-{AP}_{PN\;initial}\right)}\right]\times \frac{1}{\Delta t}$$

Where PN is N concentration in the sample, AP is ^15^N enrichment (atom% ^15^N) of the sample or substrate (N_2_) pool at the beginning (initial) and end (final) of incubation; Δt is the length of incubation. N_2_ fixation rate expressed in terms of fixation of molecular N_2_ to organic material was then calculated:

$$N_{2}\;fixation\;rate\;(mol\;N_{2}\;g^{-\;1}\;h^{-1})=V(t^{-1})\times \frac{{PN}_{final}}{2}$$

### Isotope and tissue nutrient analyses

Tissue N and P concentration of shoot and rhizome samples were assessed. Stable isotopes of N were measured by continuous flow isotope ratio mass spectrometry using a PDZ Europa ANCA-GSL elemental analyzer interfaced to a PDZ Europa 20–20 isotope ratio mass spectrometer (Sercon Ltd., Cheshire, UK). Dried samples containing approximately 20–150 μg N (2-3mg of sample) were packaged in tin capsules (Elemental microanalysis, Manchester, MA) and combusted at 1000°C in the elemental analyzer. The ratio of ^15^N/^14^N (R15) was measured for the sample and for an injection of standardized N_2_ gas introduced into the mass spectrometer in each sample cycle. The δ^15^N was calculated from:

$$\delta^{15}N=\left[(\frac{R15sample}{R15standard})-1\right]\times 1000$$

and expressed on “per mil” basis.

Total P was measured spectrophotometrically using ascorbic acid reduction of phosphomolybdate complex after combustion and consequent acid digestion [43].

### Proportion of N derived from N_2_-fixation

Percent of N derived from N_2_-fixation was calculated for the experiment using the two-endmember linear mixing model [44] formulated from the mass balance equations:

$$\delta_{M}=f_{A}\delta_{A}+f_{B}\delta_{B}$$

$$1=f_{A}+f_{B}$$

$$For\;source\;A:\;f_{A}=\frac{\left(\delta_{M}-\delta_{B}\right)}{\left(\delta_{A}-\delta_{B}\right)}$$

where δ_M_, δ_A_ and δ_B_ represent the mean isotopic signatures of the mixture M and sources A and B respectively, in our case mixture M was the isotopic signature of plants at the end of the experiment, source A had the isotopic signature of 67.74‰ (δ^15^N of the nitrate-N); source B was 0‰ (δ^15^N of the N from the air). For the calculation of the means and confidence intervals we used the ISOERROR 1.04 Excel spreadsheet by Phillips and Greg [44].

### Quantifying the presence of diazotrophs in rhizomes and roots by qPCR

The presence of diazotrophs was assessed in Atitlán rhizome samples from 2011 and in selected root and rhizome samples from the cultivation experiment. Total DNA was extracted from *Schoenoplectus* rhizomes or roots using the Power Soil DNA Isolation Kit (Mo Bio Laboratories), according to manufacturer’s instructions. DNA quality and quantity was determined by electrophoresis and total DNA concentration in the samples was measured fluorometrically. Total bacteria (16S rDNA gene) were quantified using universal primers 341f (CCT ACGGGAGGCAGCAG) and 515r (ATTCCGCGGCTGGCA) as described by [45]. The qPCR reactions were set up using the FastStart Universal SYBR Green Master Mix (Roche). First denaturation at 95°C for 10 min was followed by 30 cycles of denaturation (95°C, 45s), annealing (60°C, 30s), and extension (72°C, 30s). For the quantification of diazotrophs (*nifH)* the IGK3 (GCIWTHTAYGGIAARGGIGGIATHGGIAA) and DVV (ATIGCRAAICCICCRCAIACIACRTC) primers were used [46]. First denaturation at 95°C for 10 min was followed by 40 cycles of denaturation (95°C, 15s), annealing (58°C, 30s), and extension (72°C, 60s). Standard curves were obtained with serial 10 fold dilutions of a known amount of amplicon of the 16S rDNA and *nifH* genes, respectively. Amplicons were prepared from genomic DNA of *E*.*coli* for 16S RDNA gene and from *Methylocystis heyeri* for *nifH* gene. Each extraction, no-template control, and standard curve dilution was replicated three times. Average copy number per μl of reaction qPCR mixture was converted into copies of the gene per ng of total extracted DNA. Standard deviation was determined by the StepOne Software v2.3 (Thermo Fisher Scientific). According to threshold cycles (C_T_) of standards and the NTC values, a detection limit of approximately 10 to 100 gene copies per assay was achieved for *nifH* and 16S rDNA quantification, which corresponds to 10^2^ to 10^3^ gene copies per gram of dry rhizome or root dry weight.

### Data analyses

Because most of the data sets exhibited variance heterogeneity (F_max_-test; [47]), we used non-parametric Kruskal-Wallis Test (Statview 5 software package), to evaluate the effect of different treatments and the effects of clones (separate for each treatment) and *Schoenoplectus* variety (separate for each treatment) on response variables. For the same reason, non-parametric Mann-Whitney U-test was used instead of Student’s t-test to test the difference between means of two samples.

## Results

### Preliminary survey

Tests of N_2_-fixation capability of epiphytic and endophytic diazotrophs, providing preliminary data for future research revealed that four randomly selected *Schoenoplectus californicus* clones from our clone collection, as well as the same species from two locations in the Central Valley of California did display nitrogenase activity. The nitrogenase activity for rhizomes ranged from 13.0 to 40.9 nmol C_2_H_4_ gDW^-1^d^-1^, while root-associated activity varied from 523 to over 2000 nmol C_2_H_4_ gDW^-1^d^-1^ (Table 3). No nitrogenase activity was found in any of the shoots (data not shown). *Schoenoplectus acutus* and *Typha domingensis* both showed nitrogenase activity related to roots, but contrary to *S*. *californicus*, we did not find any nitrogenase activity in rhizome tissue of these two species.

**Table 3:** Means ± standard deviation of nitrogenase activity measured as ethylene production of of epiphytic (roots) and endophytic (rhizomes) diazotrophs from *Schoenoplectus californicus*, *S*. *acutus* and *Typha domingensis*.

Species	Location	Rhizome		Root	
		(nmol C_2_H_4_ gDW^-1^ d^-1^)			
*S*. *californicus*	Pucon, Chile	35.1	± 7.8	1085.4	± 536.5
*S*. *californicus*	Puente, Chile	15.1	± 4.2	523.4	± 318.5
*S*. *californicus*	San Juan, Guatemala	40.9	± 17.0	2012.3	± 973.1
*S*. *californicus*	Saramiento, Argentina	11.7	± 2.1	na	
*S*. *californicus*	Rio Vista, California	13.0	± 4.5	515.7	± 214.8
*S*. *californicus*	Lindsey Slough, California	14.9	± 9.4	884.6	± 390.7
*S*. *acutus*	Cosumnes, Central Valley, California	1.5	± 0.3	112.2	± 884.6
*Typha domingensis*	Cosumnes, Central Valley, California	0.0		3465.8	± 1712.4
*Typha domingensis*	Deep marsh, Belize	0.0		968.0	± 368.5

The first four samples are from plants originating in Central and South American locations and cultivated in the common garden in Davis, CA.

### Case study of BNF associated with *Schoenoplectus californicus* populations at Lake Atitlán

Nitrogenase activity of both rhizome and root associated diazotrophs was found in all tested samples (Table 4). Due to the large variability, only the endophytic rhizome fixation was significantly different between polluted *vs*. unpolluted sites, with mean values of 14 and 32 nmol C_2_H_4_ gDW^-1^d^-1^, respectively (Table 4). The nitrogenase activity associated with roots was on average more than three times higher in the unpolluted zones, although this difference was not statistically significant. There did not seem to be any trend related to time (August 2010 –rainy season *vs* February 2011 –dry season). No correlation was found between rhizome and root N_2_-fixation.

**Table 4:** Means ± standard deviations of nitrogenase activity of epiphytic (roots) and endophytic (rhizomes) diazotrophs measured as ethylene production (nmol C_2_H_4_ gDW^-1^ d^-1^), shoot and rhizomes δ^15^N, and tissue N and P of *Schoenoplectus californicus* from unpolluted and polluted locations in the littoral of Lake Atitlán, Guatemala.

Location	Date	Rhizome		Root		Shoot δ15N	Rhizome δ15N	Shoot N	Stem P
		(nmol C2H4 gDW-1 d-1^)^				‰	‰	%	%
Unpolluted									
Isla	2010	47	± 13	209	± 34	-4.2	na	1.8	0.13
Isla	2011	22	± 10	9562	± 6395	0.9	-0.2	1.8	0.16
San Marcos	2010	43	± 27	3012	± 2806	0.3	na	1.5	0.1
San Marcos	2011	12	± 3	348	± 264	2	1.6	2	0.16
San Pedro	2011	36	± 18	157	± 36	-8.2	-5.8	1.7	0.12
**Mean**		**32**		**2658**		**-1.8**	**-1.5**	**1.8**	**0.13**
Polluted									
San Lucas	2010	16	± 14	1374	± 973	na	na	1.8	0.13
San Lucas	2011	14	± 10	151	± 35	-3	-0.3	1.8	0.17
Santiago	2011	18	± 12	11	± 3	-5.1	-4.2	2.1	0.16
Panajab	2011	9	± 2	1311	± 230	0.7	0.9	1.8	0.14
**Mean**		**14**		**712**		**-2.5**	**-1.2**	**1.9**	**0.15**
*P value*	* *	***0*.*03***	*** ***	*NS*	* *	*NS*	*NS*	*NS*	*NS*

2010 rainy season, 2011 dry season. P-values (Mann-Whitney U-test) indicate the significance of differences between the means for polluted versus non-polluted locations. na = not available; NS = not significant.

**Fig 2:**
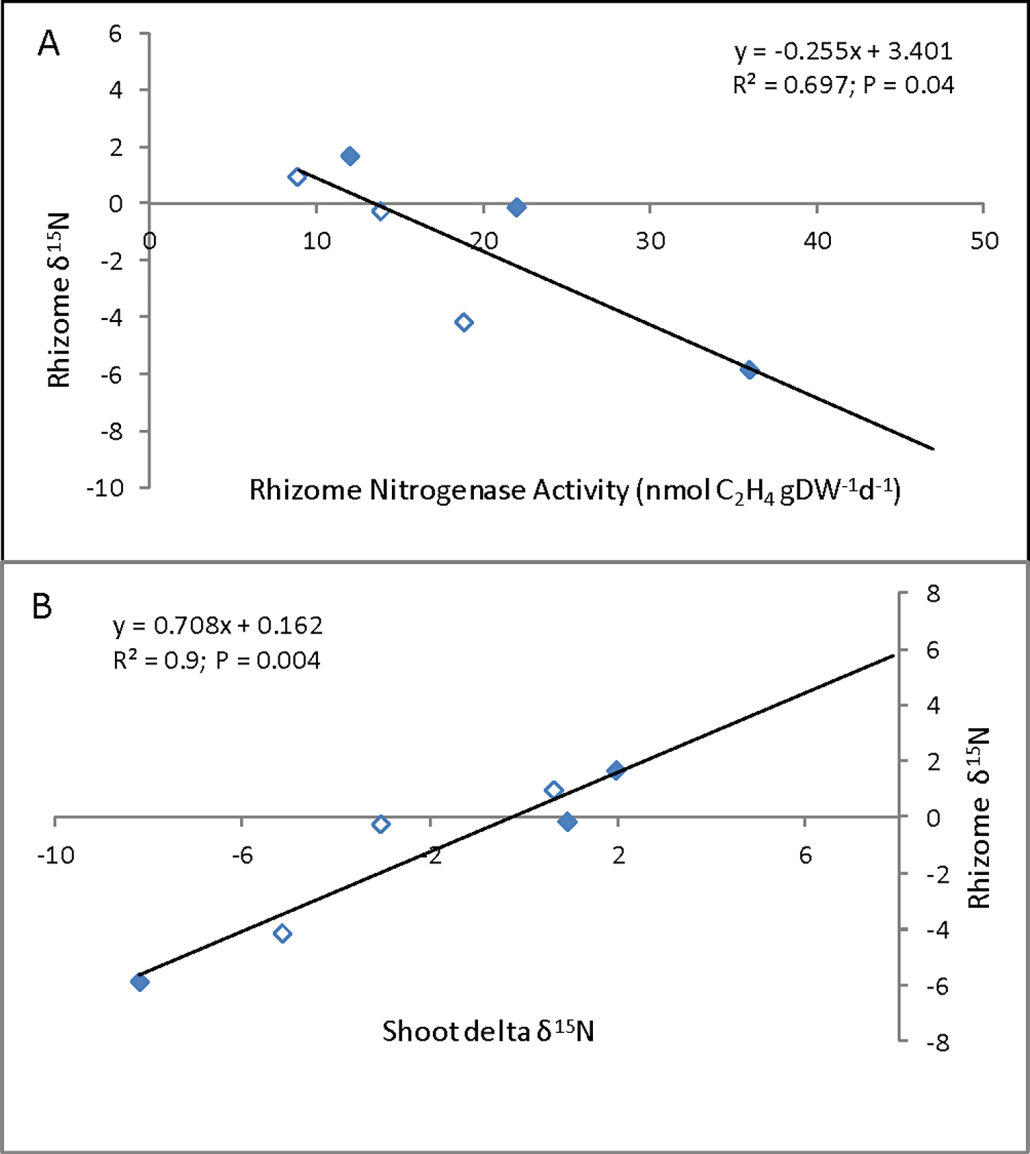
Relationship between rhizome nitrogenase activity and rhizome δ^15^N (A) and shoot and rhizome δ^15^N (B) in *Schoenoplectus californicus* from Lake Atitlán; full diamond–unpolluted sites, empty diamonds–polluted sites. Each nitrogenase activity value is a mean of 3–4 replicates, isotope data were measured on pooled samples. Nitrogenase activity expressed as ethylene production (nmol C_2_H_4_ gDW^-1^ d^-1^).

Delta ^15^N of shoots ranged from 2.0 ‰ to -8.2 ‰ averaging -2.1 ‰; the rhizome δ^15^N ranged from 1.6 ‰ to -5.8 ‰ averaging -1.3 ‰ (Table 4). There was correlation between rhizome nitrogenase activity and rhizome δ^15^N (R^2^ = 0.69; P = 0.04; Fig 2A), but not the shoot δ^15^N; this despite the fact that shoot δ^15^N and rhizome δ^15^N were well correlated (R^2^ = 0.9; P = 0.004; Fig 2B).

Diazotrophs (based on the *nifH* gene presence) were detected in all analyzed samples (Table 5). The proportion of *nifH* gene copies among the total bacterial DNA was low, but well above the method detection limit. It varied from 0.01 to 0.04% (Table 5). While there was a trend of increasing nitrogenase activity with increasing *nifH* proportion, the correlation was not significant, however, it became significant when all availbale data were included (see the text in the Cultivation experiment subchapter).

**Table 5:** Nitrogenase activity of epiphytic (roots) and endophytic (rhizomes) diazotrophs measured as ethylene production (nmol C_2_H_4_ gDW^-1^ d^-1^) associated with *Schoenoplectus californicus* from unpolluted and polluted locations in the littoral of Lake Atitlán, Guatemala and with its selected varieties in different nutrient treatments of the cultivation experiment.

	Roots/ Rhizomes	Location/ Treatment	16S rDNA copies ng^-1^ DNA	nif gene copies ng^-1^ DNA	Proportion *nifH* (%)	Nitrogenase activity nmolC_2_H_4_ gDW^-1^ d^-1^
**Atitlán Case**	rhizomes	Isla	1812612	259	0.0143	21
**Study**	rhizomes	San Lucas	7698841	831	0.0108	6.5
	rhizomes	San Lucas	4196620	515	0.0123	9.9
	rhizomes	Panajab	7485182	925	0.0124	8.6
	rhizomes	Panajab	9327930	1257	0.0135	11.9
	rhizomes	San Martin	763744	58	0.0076	9.8
	rhizomes	San Martin	7668365	2297	0.0300	10.8
	rhizomes	Santiago	6269800	2273	0.0362	14.6
	rhizomes	San Pedro	9361064	2508	0.0268	47
	rhizomes	San Pedro	8266368	1910	0.0231	16
**Cultivation Experiment**						
Guillermo	roots	zero N	2241591	1740	0.0776	96.4
Guillermo	roots	Low N	1743083	1209	0.0694	597.9
Guillermo	roots	High N	2571891	4556	0.1771	216.3
Puente	roots	zero N	1283339	6540	0.5096	307.9
Puente	roots	zero N	1984252	1502	0.0757	597.6
Puente	roots	Low N	1504478	2501	0.1662	215.6
Puente	roots	High N	1941164	2610	0.1345	966.1
Puente	roots	High N	2335219	1251	0.0536	600.9
Rockefeller	roots	zero N	1759754	3556	0.2021	2328.1
Rockefeller	roots	Low N	2279659	801	0.0351	115.7
Rockefeller	roots	High N	3329109	2010	0.0604	1672.4
Ensenada	roots	zero N	2610378	1832	0.0702	455.2
Ensenada	roots	zero N	2803902	4420	0.1576	3252.9
Ensenada	roots	Low N	2156471	1298	0.0602	234.6
Ensenada	roots	Low N	3830354	5711	0.1491	2327.4
Ensenada	roots	High N	2370427	8624	0.3638	6087.8
Guillermo	rhizomes	High N	1848001	31	0.0017	46
Puente	rhizomes	High N	3726492	87	0.0023	110
Rockefeller	rhizomes	zero N	2542134	144	0.0057	320
Rockefeller	rhizomes	High N	2648460	48	0.0018	55

The presence of *nifH* genes is expressed as copies per ng DNA or as a proportion of diazotrophs (*nifH* gene copies) in the total bacterial DNA pool (%).

### Cultivation experiment

#### Growth response to nitrogen treatments

Biomass of shoots, rhizomes, and roots as well as cumulative shoot length was recorded at the end of the experiment (87 days). In each treatment, all these response variables were closely correlated (R^2^ >0.9), thus we present total dry mass only (Table 6). As expected, the effect of the N treatment was highly significant; the plants in zero N treatment grew very slow with the average biomass per pot of 8.8 g, however, they did form new healthy shoots. The low N and high N treatments resulted in correspondingly higher biomass with 14.1 and 54.4 g per pot, respectively. The *tere* variety produced significantly less biomass than *cali* variety in each of the N treatments. Plants in zero N treatment invested more growth effort into roots as documented by a low shoot/root ratio of 0.69 as compared to 1.63 and 2.68 for low N and high N treatments respectively. (Note, that “root” in S/R ratio stands for both roots and rhizomes). The tissue N content was low in zero N and low N treatment, 0.60 and 0.85% respectively, and significantly higher, 1.71%, for high N treatment. In low and high N treatment, the *tere* variety contained more N in shoot tissue than *cali*. Nearly all the N added to the low N treatment pots was recovered in the biomass of plants in the low N treatments.

**Table 6:** Means ± standard deviations of response variables of *Schoenoplectus californicus* to nitrogen treatment.

	Clone	DW		S/R		N%		N-fix Rhizome		N-fix Root		N_dfa_		δ^15^N	
**Z**	GU	3.9	± 1.4	0.19	± 0.18	0.56	± 0.20	48.7	± 9.8	67	± 25	N/A		4.2	± 1.9
	PT	6.8	± 2.2	0.79	± 0.08	0.47	± 0.03	22.7	± 8.3	420	± 155	N/A		4.7	± 1.2
**E**	EN	5.0	± 0.5	0.45	± 0.29	0.66	± 0.30	82.2	± 44.6	1593	± 1469	N/A		1.5	± 0.8
	CC	11.0	± 3.5	0.57	± 0.18	0.56	± 0.12	88.0	± 36.7	254	± 89	N/A		4.7	± 0.6
**R**	RW	12.1	± 2.8	0.82	± 0.22	0.68	± 0.11	37.67	± 72.3	1384	± 1334	N/A		2.9	± 0.4
	SJ	9.8	± 1.7	0.44	± 0.08	0.72	± 0.17	84.7	± 27.1	909	± 226	N/A		3.4	± 0.4
**O**	HU	9.2	± 1.8	1.21	± 0.45	0.54	± 0.03	162.2	± 96.5	608	± 590	N/A		0.2	± 0.2
	PU	12.9	± 5.0	0.45	± 0.02	0.60	± 0.13	128.3	± 50.3	1136	± 42	N/A		0.9	± 0.4
		**8.8**		**0.69**		**0.60**		**124.2**		**745.1**				**2.8**	
	GU	7.5	± 0.5	1.5	± 0.70	1.30	± 0.10	68.4	± 61.3	341	± 224	14.7	± 0.9	58.4	± 0.8
**L**	PT	11.5	± 1.7	1.6	± 0.20	0.80	± 0.09	49.6	± 11.2	238	± 133	17.8	± 3.6	56.5	± 2.3
	EN	9.6	± 0.1	1.5	± 0.90	1.01	± 0.19	193.4	± 43.3	1632	± 984	26.1	± 10.0	50.5	± 6.6
**O**	CC	15.1	± 2.9	1.6	± 0.50	0.75	± 0.22	43.2	± 7.0	223	± 142	26.5	± 3.7	51	± 2.4
	RW	13.9	± 3.0	2.2	± 0.40	0.88	± 0.08	77.9	± 25.8	360	± 291	24.2	± 4.9	51.8	± 2.9
**W**	SJ	13.1	± 1.4	1.3	± 0.10	0.75	± 0.06	99.8	± 52.0	934	± 116	34.1	± 18.6	45.7	± 12.0
	HU	19.5	± 2.4	2.1	± 0.40	0.75	± 0.19	90.3	± 0.2	361	± 23	22.6	± 1.7	52.5	± 1.2
	PU	22.2	± 2.1	1.2	± 0.20	0.58	± 0.07	261.6	± 32.5	828	± 358	30.6	± 6.9	47.3	± 4.6
		**14.1**		**1.63**		**0.85**		**110.6**		**614.6**		**23.7**		**51.7**	
**H**	GU	11.9	± 5.3	3.0	± 0.70	2.31	± 0.12	83.9	± 33.3	312	± 246	1.5	± 1.6	66.9	± 1.2
	PT	32.3	± 7.3	3.1	± 1.30	1.91	± 0.22	112.8	± 25.2	889	± 258	2.7	± 2.4	66.5	± 2.3
**I**	EN	35.5	± 9.3	2.8	± 0.70	1.87	± 0.29	217.3	± 153.6	4219	± 1621	2.7	± 2.7	66.3	± 2.4
	CC	65.8	± 11.5	2.0	± 0.10	1.79	± 0.17	137.9	± 40.2	2256	± 1101	0.5	± 0.9	67.9	± 0.9
**G**	RW	43.9	± 4.3	3.1	± 0.90	1.60	± 0.13	73.9	± 62.8	2779	± 972	2.6	± 3.6	66.9	± 3.5
	SJ	61.8	± 2.4	2.6	± 0.30	1.50	± 0.08	171.4	± 10.4	5380	± 29	6.8	± 8.3	63.4	± 5.3
**H**	HU	74.0	± 9.5	3.1	± 0.20	1.22	± 0.08	185.8	± 190.8	3912	± 2113	1.5	± 2.3	67	± 1.8
	PU	111.1	± 19.0	1.8	± 0.20	1.48	± 0.08	460.9	± 32.4	2922	± 1387	2.8	± 0.8	65.8	± 0.6
		**54.4**		**2.68**		**1.71**		**180.5**		**2833.7**		**2.1**		**66.3**	
***Effect***	* *	* *	* *	* *	* *	* *	* *	* *	* *	* *	* *	* *	* *	* *	* *
TREATMENT		*0*.*001*		*0*.*0001*		*0*.*0001*		*0*.*08*		*0*.*0001*		*0*.*0001*		*0*.*0001*	
CLONE zero N		*0*.*05*		*0*.*07*		*NS*		*0*.*02*		*0*.*05*		*-*		*0*.*01*	
CLONE low N		*0*.*01*		*NS*		*0*.*05*		*0*.*10*		*NS*		*0*.*05*		*0*.*08*	
CLONE high N		*0*.*004*		*NS*		*0*.*01*		*0*.*10*		*0*.*03*		*NS*		*NS*	
VAR zero N		*0*.*001*		*NS*		*0*.*07*		*0*.*002*		*NS*		*-*		*0*.*10*	
VAR low N		*0*.*0004*		*NS*		*0*.*01*		*NS*		*NS*		*0*.*01*		*0*.*01*	
VAR hig hN		*0*.*0001*	* *	*NS*	* *	*0*.*01*	* *	*NS*	* *	*0*.*03*	* *	*NS*	* *	*NS*	

Zero = no nitrogen added; Low = 120 mg N per pot; High = 1200 mg N per pot; DW = dry mass at the end of the experiment in g per pot; S/R = aboveground to belowground biomass ratio; N% = aboveground tissue N content; N-fix Rhizome = endophytic N_2_ fixation and N-fix Root = epiphytic N_2_ fixation, both measured as ethylene production (nmol C_2_H_4_ gDW^-1^d^-1^); N_dfa_ % = proportion of N in the shoots from N fixation; shoot δ^15^N in ‰ at the end of the experiment. Guiellermo, GU, Puente, PT, and Ensenada, EN, are clones of variety *tereticulmis*; Copacabana, CC, Rockefeller, RW, San Juan, SJ, Huanchaco, HU, Puno, PU, are clones of variety *californicus*.

#### Response of nitrogenase activity to nitrogen treatments

**Fig 3:**
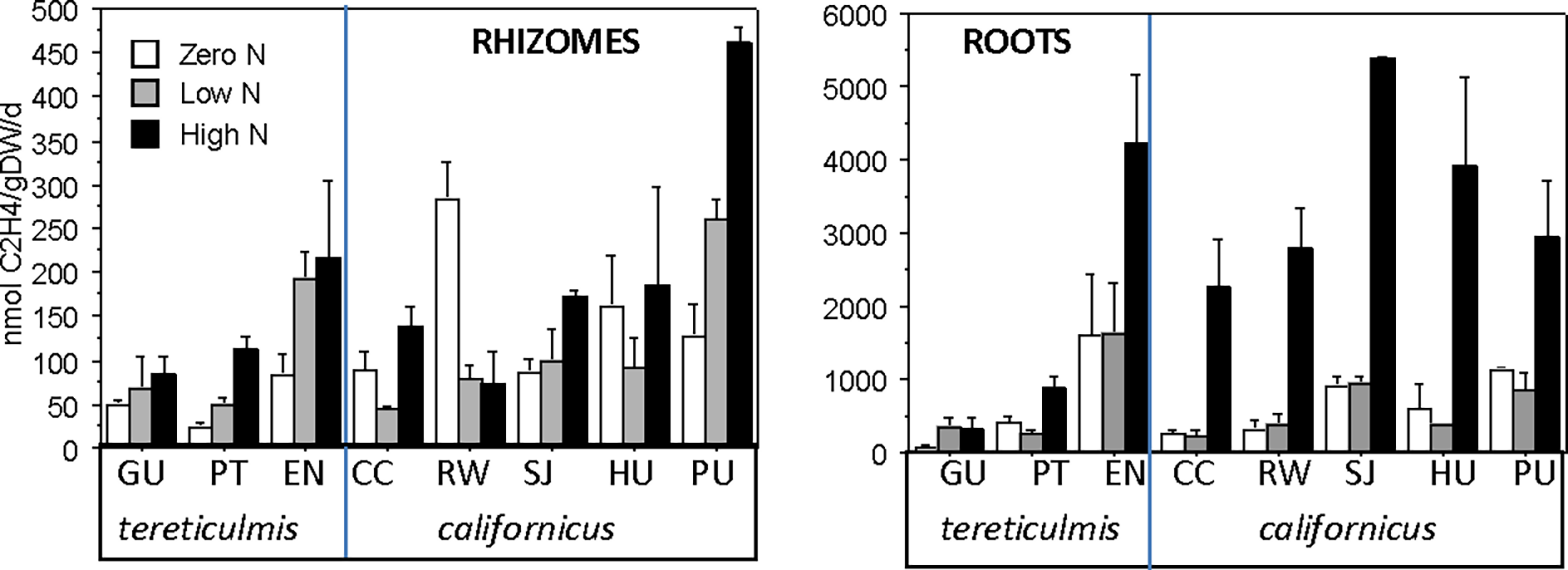
Nitrogenase activity expressed as ethylene production (nmol C_2_H_4_ gDW^-1^ d^-1^) of endophytic bacteria (RHIZOMES) and epiphytic bacteria (ROOTS) of *Schoenoplectus californicus* in the experiment. X-axis: Clones Guillermo, GU, Puente, PT and Ensenada, EN are of variety tereticulmis; Copacabana, CC, Rockefeller, RW, San Juan, SJ, Huanchaco, HU, and Puno, PU are clones of variety californicus. The error bars indicate the standard error of the mean; n = 3.

We found evidence of endophytic (rhizome) N_2_-fixation in all treatments and all clones. The activity fluctuated considerably, ranging from 22.7 nmol C_2_H_4_ g DW^-1^d^-1^ to 460 nmol C_2_H_4_ g DW^-1^d^-1^ (Table 6, Fig 3). There were no differences between zero and low N treatments because of the large variability, but rhizome nitrogenase activities in low and high N treatment were closely correlated (R^2^ = 0.81; P = 0.002). Rhizome fixation was significantly lower in the *tere* variety. Root (epiphytic) nitrogenase activity was also highly variable and did not differ between zero and low N treatment, while it was on average about 4x higher in high N treatment. Root nitrogenase activity was positively correlated among all three treatments. Endo- and epiphytic fixations were not correlated except for the low N treatment (positive correlation; R^2^ = 0.51; P = 0.05).

#### Proportion of N from N_2_-fixation (N_dfa_)

**Fig 4:**
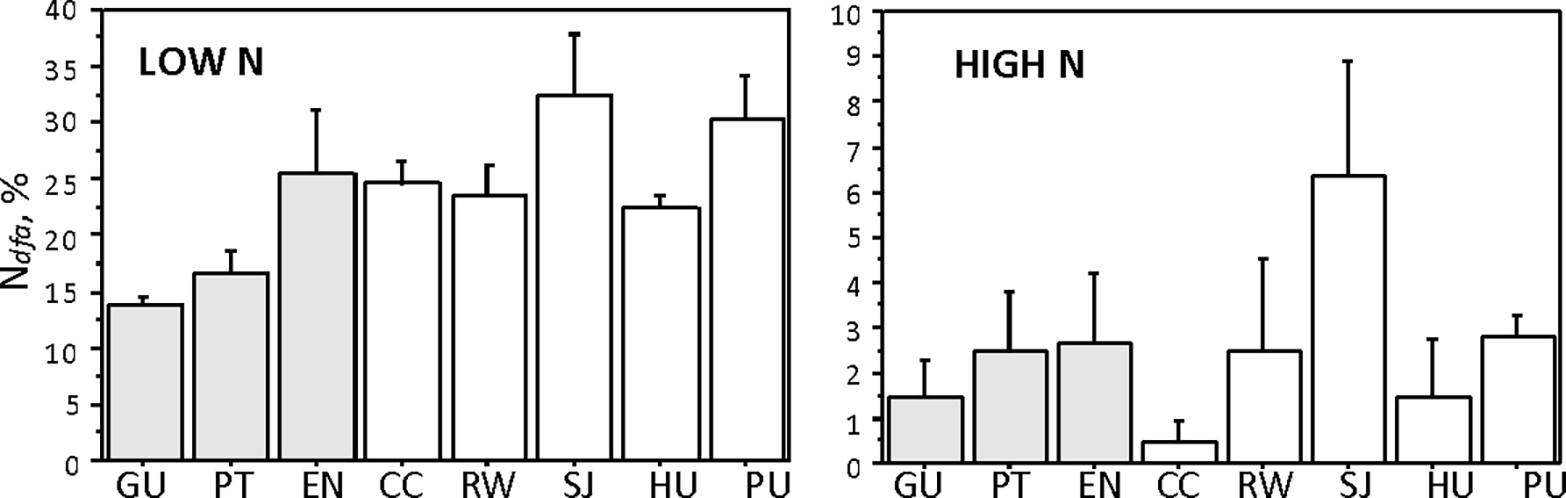
The proportion of N derived from N_2_ fixation (N_dfa_). X-axis: Clones Guillermo, GU, Puente, PT and Ensenada, EN are of variety *tereticulmis*; Copacabana, CC, Rockefeller, RW, San Juan, SJ, Huanchaco, HU, and Puno, PU are clones of variety *californicus*. Note the differences in the y-axes. The error bars indicate the standard error of the mean (n = 3).

For the low N and high N treatments, we used the mixing model to calculate the proportion of N derived from N_2_-fixation (N_dfa_). In the low N treatment, we found differences in the contribution to the plant N content among clones from different locations ranging from 13.8% for Guillermo to 32.5% for San Juan (Fig 4). Variety *cali* utilized the N from N_2_-fixation significantly more than *tere* (N_dfa_ 26.3% and 18.6%, respectively; Mann-Whitney U-test; P = 0.01). In the high N treatment the N_dfa_ was very low (0.5 to 6.4%) and the differences between clones were not significant, although San Juan location, SJ, had the highest proportion of N from N_2_-fixation in both treatments. No differences between varieties were found in the high N treatment.

**Fig 5:**
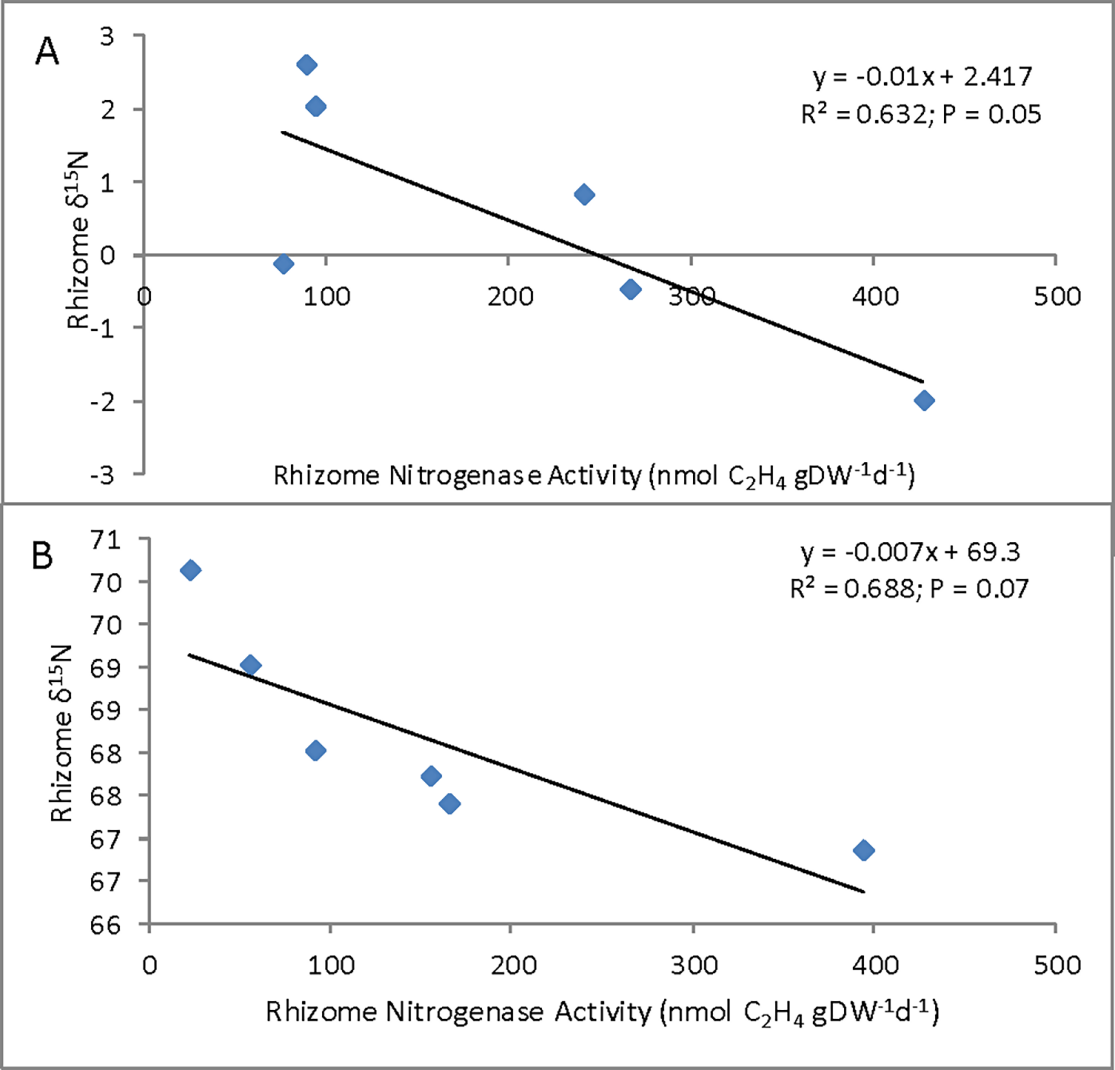
Relationship between rhizome nitrogenase activity expressed as ethylene production (nmol C_2_H_4_ gDW^-1^ d^-1^).and rhizome δ^15^N in *Schoenoplectus californicus* from the cultivation experiment zero N treatment (A) and high treatment (B). Each nitrogenase activity value is a mean of 3 replicates, isotope data are measured on pooled samples.

Similarly to the Atitlán case study, there was a negative correlation between rhizome nitrogenase activity and rhizome δ^15^N in both zero and high N treatments (Fig 5). Not enough data on rhizome δ^15^N were available to calculate correlations for low N treatment. Rhizome and shoot δ^15^N values were significantly positively correlated in zero treatment (R^2^ = 0.66; P = 0.05), while non-significant positive trend was found in high N treatment (R^2^ = 0.43; P = 0.15).

**Fig 6:**
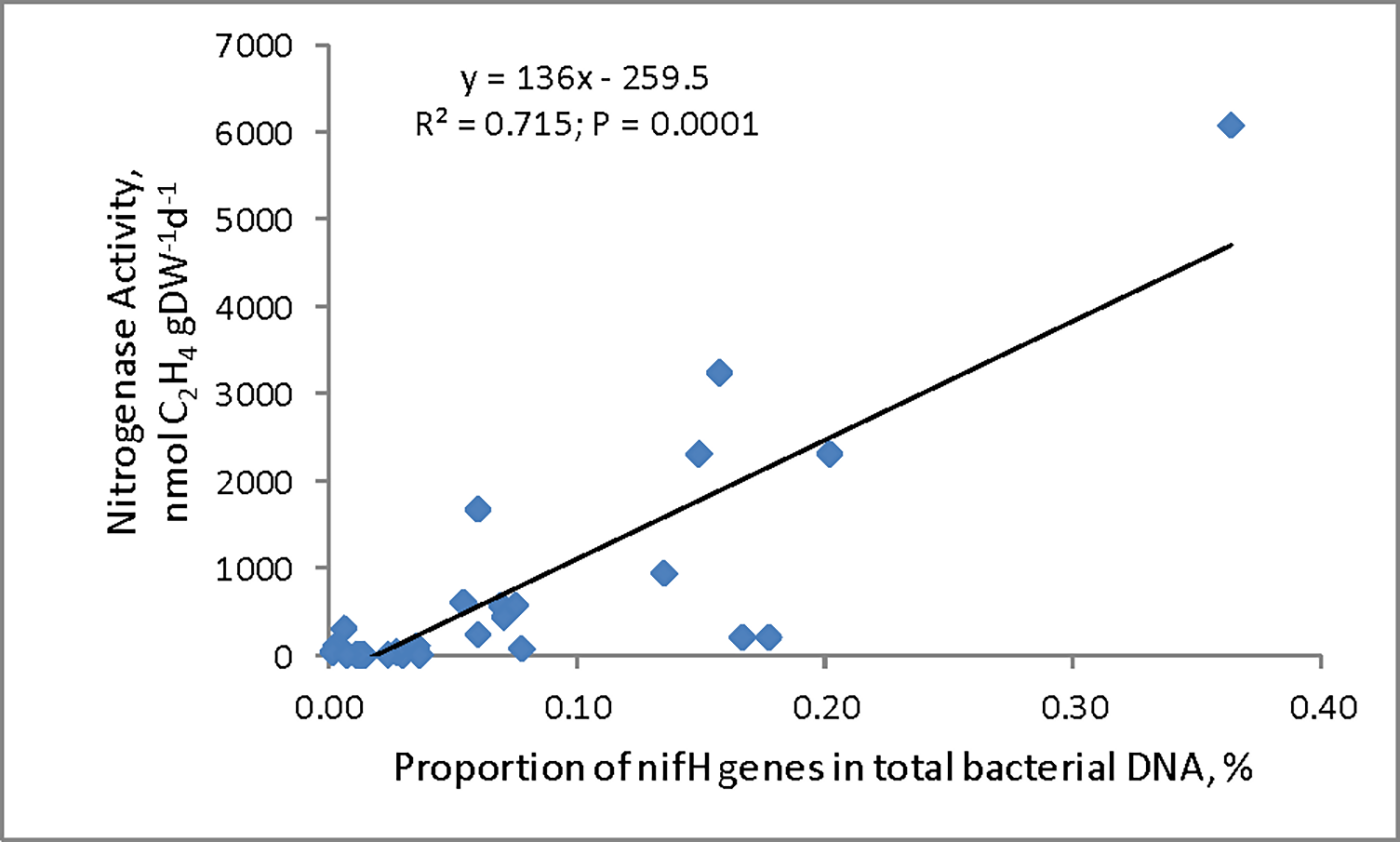
Relationship between nitrogenase activity expressed as ethylene production (nmol C_2_H_4_ gDW^-1^ d^-1^) and proportion of *nifH* genes in total bacterial DNA. Combined data from Atitlán case study and the cultivation experiment.

On average 40x more *nifH* gene copies were found in the root material than in the rhizomes (Table 5). When the nitrogenase activity was plotted against the proportion of *nifH* gene for all samples (rhizomes from the Atitlán case study and roots and rhizomes from the cultivation experiment), the correlation was highly significant (Fig 6).

#### Calibration of ARA through ^15^N_2_ reduction assay

In both rhizomes and roots, the nitrogenase activity measured by ARA was correlated closely to the ^15^N_2_ reduction (rhizomes: y = 2.785x; r^2^ = 0.98, roots: y = 2.38x; r^2^ = 0.87) denoting that the C_2_H_4_ reduction: N_2_ reduction ratio was 2.78: 1 and 2.38: 1 for rhizomes and roots respectively.

## Discussion

Our hypothesis that endophytic and epiphytic diazotrophs are associated with rhizomes and roots of *Schoenplectus californicus* throughout its distributional range and that the plant is capable of utilizing the fixed N has been confirmed by several lines of evidence (nitrogenase activity, presence of *nifH* gene, and δ^15^N data).

There is very little information available on N_2_-fixation associated with members of the Cyperaceae family and none that would relate to endophytic N_2_-fixation. Rhizosphere diazotrophs associated with *Schoenoplectus americanus*, a close relative of *S*. *californicus*, were reported to fix 367 ± 46 ng ^15^N per plant per hour [9]. A direct comparison is difficult because of differences in the experimental setting, but using our C_2_H_4_: N_2_ ratio of 2.38 (see results) and shoot: root ratio of ~ 1 [9], their value would translate to some 20,000 nmol C_2_H_4_ g^-1^d^-1^_,_ i.e., an order of magnitude higher value compared to our data. This is potentially explainable by the fact that Dakora and Drake used intact plants for measurements and thus diazotrophs had an unrestricted access to root carbon exudates (see below). Root associated diazotroph activity in the same range as *Schoenoplectus californicus* has been reported for *Cyperus papyrus*, a dominant species of tropical swamps in Africa [16]. *Eleocharis* spp. from marshes in Belize displayed root associated N_2_ fixation on the order of 3000 to 4000 nmol C_2_H_4_ g^-1^d^-1^ under conditions unlimited by carbon [11]. As for the wetland plants other than Cyperaceae, Eckard and Biesboer [14] reported the nitrogenase activity of 217 and 226 C_2_H_4_ g^-1^d^-1^ for *Typha latifolia* and *T*. *angustifolia*, respectively, and they concluded that populations of *Typha* may receive as little as 1–2% of their annual N requirement from N_2_-fixation.

### Are there any differences between N_2_-fixation of roots (epiphytic) and rhizomes (endophytic) associated diazotrophs?

Our prediction that the root associated N_2_-fixation will be higher than endophytic N_2_-fixation was correct. In the Atitlán data set as well as in the cultivation experiment, the epiphytic N_2_-fixation was 6 to 60 times higher than the endophytic N_2_-fixation. This is in agreement with the general consensus that endophytic bacteria occur at lower population densities than surface associated epiphytic bacteria [22]. This difference can also result from the way we present the results, i.e., as nitrogenase activity per grams of dry weight. Because of their bulky structure, *Schoenoplectus* rhizomes have relatively large proportion of a “ballast” biomass, while fine roots provide a large surface area for diazotrophs to attach to. In the case of *S*. *californicus* with its extensive root structure, it seems possible that the plants benefit more from the epiphytic bacteria, but at this point it is still just a speculation. We assume that the diazotrophs in the root samples are epiphytic, but since we did not sterilize the roots, we cannot really exclude the possibility of endophytic root diazotrophs being present.

### The importance of carbon limitation

The presence of a constant C supply as energy source is an important criterion to be satisfied for the diazotrophs [10, 48]. It is quite probable that the N_2_-fixation of both rhizomes and roots in our measurements was underestimated, because in our experimental setting the diazotrophs on excised roots/in rhizomes did not have access to the natural and continuous input of C. A recent trial showed that, with the addition of glucose, the root fixation was on average 6x higher (Rejmánková, unpublished data). Dalton et al. [37] reported a 9-fold increase in the nitrogenase activity associated with roots of *Elymus mollis* after the roots have been immersed in 1% glucose prior to the incubation treatment. Lower N_2_ fixation was reported on excised roots of *Cyperus papyrus* compared to fixation on intact roots [16] and the C limitation has been further demonstrated by other authors [22, 49]. Based on the increased N_2_ fixation caused by the enhanced photosynthate supply to root-associated diazotrophs, Dakora and Drake [9] suggest that, as in legumes, a direct relationship exists between C supply and N yield from N_2_-fixation associated with the *Cyperaceae*. The relationship between C exudates and the diazotroph activity associated should be explored further.

### N_dfa_

Estimates of nitrogenase activity provide important information but it may not reflect the amount of N actually utilized by plants. The proportion of N a plant obtains from N_2_-fixation is more meaningful. In our low N addition treatment, the N_dfa_ ranged from 13.8% to 32.5% and was higher in the *cali* variety as compared to the *tere* (26.3% and 18.6%, respectively). The available data on N_2_-fixation contribution vary greatly and generally seem to be higher in plants artificially inoculated with a single strain or a mix of N-fixing bacteria. Field studies with “wild”, non-inoculated plants report N_2_-fixation contributions ranging from 0 to 30% [13, 50]. The “engineered” plants, on the other hand, often obtain more than 50% of N from BNF (e.g., sugarcane: 34.8–58.8% [51]; rice variety BAS-370 close to 70% [52]; *Pennisetum* 50% [12]; poplar 65% [20], etc.). Presently, *S*. *californicus* is an important economical plant in several regions, where it is used for boat construction or/and mat weaving [26], but the demand is still covered by natural production. Should the larger demand for this species occur, inoculation trials with endophytic diazotrophs may become important.

### Constitutive endophytic N_2_-fixation

N_2_-fixation with its very high energy requirement should, theoretically, down-regulate where there is high N availability in the soil [29]. In our experiment, we surprisingly found a larger nitrogenase activity in both rhizomes and roots in high N treatments than in zero N treatment. This could be potentially explained by localized depletion of available N in parts of the rhizosphere. Towards the end of the experiment, plants in high N treatment were growing vigorously and some could have used most/all of added N. This would activate diazotrophs, which would likely perform well due to high quality carbon released as exudates from well growing plants. It would be in principle similar to what Hedin et al. [53] call the nitrogen paradox, where in the case of tropical forests, BNF from free living diazotrophs can occur in N-limited areas of the forest separated from areas of abundant N, allowing N_2_ fixation to continue in these specific areas, despite the ecosystem being N-rich as a whole. Field data from Atitlán indicated that there was a trend towards lower BNF in the rhizosphere diazotrophs and a significantly lower BNF in the endophytic diazotrophs among populations from polluted areas. This confirmed our prediction that the N_2_-fixation will be higher in the unpolluted areas, while in the enriched locations, plants will be utilizing N available in sediments.

### Are there any differences in N_2_-fixation between the two varieties of *S*. *californicus*?

The finding that *S*. *californicus* var. *californicus* consistently displays higher nitrogenase activity as well as higher N_dfa_ than the variety *tereticulmis* was unexpected. Considering that the clones used for this experiment had been in cultivation in Davis for several years prior to the experiment, the result could not have been due to priming of the original locations. It demonstrates that the taxonomic identity can have an important effect on the N acquisition strategy and is in agreement with Wurzburger and Hedin’s [54] finding that taxonomic identity was the major determinant of fixation across a broad soil P gradient in lowland tropical forests, and that soil nutrients had no or only limited influence on N_2_ fixation. The fact that one of the varieties seems to be capable of utilizing larger proportion of N from BNF may become important if there ever is a need to increase the production of this species.

### Is the presence of nifH gene a good predictor of N_2_-fixation?

The presence of *nifH* gene means that that bacterial community has the potential to perform N_2_-fixation, not necessarily that the process itself is present. Although the proportion of *nifH* gene copies among the total bacterial DNA was relatively low, in the samples from the cultivation experiment, the *nifH* gene quantity in both the root surface associated and endophytic bacteria was well correlated with the nitrogenase activity measured. The correlation was much weaker in (a much smaller) data set from the Atitlán case study. However, this correlation need not always be strong, because *nifH* genes are also present in DNA of inactive or non-living microbes (for example [55]). To the extent that *nifH* gene copy number reflects diazotroph abundance, the *S*. *californicus* strategy may be that of maintaining relatively low abundances of highly efficient diazotrophs on their root systems. Our data suggest that many of the diazotrophs on *S*. *californicus* roots or rhizomes were indeed alive and active, despite the fact that, in both types of experiments, they likely experienced a limitation by available plant-derived C. Measurements of N_2_-fixation using excised roots may underestimate the activity on intact roots. Unfortunately, other methods of assessing N_2_-fixation activity on intact roots are too technically challenging to be used routinely or under field conditions. Because of these limitations, the *nifH* gene does seem to be a good additional marker for the presence of N_2_-fixation in *S*. *californicus* associated bacteria. Considering the difference among the two *S*. *californicus* varieties, it remains to be assessed how the bacterial community composition varies among them, as well as how it varies between the endophytic and epiphytic bacteria.

### What can we learn from stable isotope signatures?

How does δ^15^N signature support our hypothesis that *S*. *californicus* utilizes N from BNF by its associated bacteria? The best answer comes from the field data from Atitlán, where the δ^15^N of *S*. *californicus* shoots averaged -2.1 ‰ (Table 4), as compared to the other, presumably non-fixing lake littoral species ranging from 3.4 ‰ (*Typha domingensis*) and 4.6 ‰ (submersed spp.) to 4.9 ‰ (*Salix humboltiana*) (Rejmánková, unpublished data). The average littoral sediment δ^15^N of 3.4 ‰ is close to the values measured in the non-fixing species. Similarly, in the field collection of the *S*. *californicus* clones, the δ^15^N clones from low N locations averaged 0.6 ‰, while in the N rich locations it was 12.0 ‰ (Table 2). Although, as many authors pointed out, the assumption that the δ^15^N of leaf tissues reflects that of the N source in the soil is not always valid [56, 57], plant isotope composition is more likely to reflect that of the N source when plant demand exceeds N supply [58],—this is clearly the case of N-limited wetlands throughout the *S*. *californicus* distributional range.

### Potential N budget

Data from our experiment demonstrated that in the low N addition treatment on average 24% N was derived from N_2_-fixation. For the SJ clone originating at Lake Atitlán, this proportion was 34%. How does this agree with a budget calculated from endophytic and epiphytic nitrogenase activity using Atitlán originated material? The average nitrogenase activities for plants from nutrient un-enriched locations were 37.1 and 2567.7 nmol C_2_H_4_ gDW^-1^ d^-1^, respectively. If we assume the average biomass of 400 g m^-2^, 300 g m^-2^, and 300 g m^-2^ for the aboveground stems, rhizomes, and roots, respectively (the biomass proportion from Castle, unpublished data), the longevity of plants one year, and the tissue N of 1.8%, then the plant would require 18g of N m^-2^ y^-1^. With the C_2_H_4_ reduction: N_2_ reduction ratio of 2.8: 1 and 2.4: 1 for rhizomes and roots, respectively (see results), the contribution of rhizosphere and endophytic N_2_-fixation would represent 19%. This value is lower than N_dfa_ from the experiment and this is assuming all fixed N is available to plants. Several reasons may be responsible: the longevity of plants may be higher; we may have underestimated the fixation by measuring it on excised roots (see above); and the conditions during the experiment in Davis may have been more favorable.

## Conclusion

Although the absolute contribution of N_2_-fixation is difficult to determine, our results show that the N budget of *S*. *californicus* is substantially subsidized by fixed N. In support of this, there have been multiple observations that throughout its range, in the areas heavily impacted by sewage inflow, *Schoenoplectus* is being outcompeted by *Typha domingensis* (Rejmánková, unpublished data). The *S*. *californicus* system represents a suitable model for future studies on the effects of non-symbiotic N_2_ fixation on the geographical distribution of plant species and varieties, plant physiology, or inter-species competition. As *S*. *californicus* is also an important plant for many native communities throughout Central and South America and is a species commonly used in constructed wetlands and wastewater treatment, the information presented in this paper may also help to improve its more applied functional roles.

## References

[1] Aerts R, Chapin FS. The mineral nutrition of wild plants revisited: A re-evaluation of processes and patterns. Advances in Ecological Research 2000; 30:1–67.

[2] Bedford BL, Walbridge MR, Aldous A. Patterns in nutrient availability and plant diversity of temperate North American wetlands. Ecology 1999; 80:2151–2169.

[3] Vance CP, Uhde-Stone C, Allan DL. Phosphorus acquisition and use: critical adaptations by plants for securing a nonrenewable resource. New Phytologist 2003; 157:423–447. 10.1046/j.1469-8137.2003.00695.x 33873400

[4] Ticconi CA, Abel S. Short on phosphate: plant surveillance and countermeasures. Trends in Plant Science 2004; 9:548–555. doi: 10.1016/j.tplants.2004.09.003 15501180 10.1016/j.tplants.2004.09.003

[5] Rejmánková E, Snyder J. Emergent macrophytes in phosphorus limited marshes: do phosphorus usage strategies change after nutrient addition? Plant and Soil 2008; 313:141–153.

[6] Jurelevicius D, Korenblum E, Casella R, Vital R L, Seldin L. Polyphasic analysis of the bacterial community in the rhizosphere and roots of *Cyperus rotundus* L. grown in a petroleum-contaminated soil. Journal of Microbiological Biotechnology 2010; 20:862–870 10.4014/jmb.0910.10012 20519908

[7] Turner TR, James EK, Poole PS. The plant microbiome. Genome Biology 2013; 14:209 doi: 10.1186/gb-2013-14-6-209 23805896 10.1186/gb-2013-14-6-209 PMC3706808

[8] Weier KL, Pittaway PA, Wildin JH. Role of N_2_-fixation in the sustainability of the ponded grass pasture system. Soil Biology and Biochemistry 1995; 27:441–445.

[9] Dakora FD, Drake BG. Elevated CO_2_ stimulates associative N_2_ fixation in a C3 plant of the Chesapeake Bay wetland. Plant and Cell Environment 2000; 23:943–953.

[10] Bürgmann H, Meier S, Bunge M, Widmer F, Zeyer J. Effects of model root exudates on structure and activity of a soil diazotroph community. Environmental Microbiology 2005; 7:1711–1724 doi: 10.1111/j.1462-2920.2005.00818.x 16232286 10.1111/j.1462-2920.2005.00818.x

[11] Šantrůčková H, Rejmánková E, Pivničková B, Snyder J. Nutrient enrichment in tropical wetlands: shifts from autotrophic to heterotrophic nitrogen fixation. Biogeochemistry 2010; 101:295–310.

[12] Reis VM, dos Reis FB Jr, Quesada DMB, de Oliveira OCA, Alves BJR, Urquiaga S et al Biological nitrogen fixation associated with tropical pasture grasses. Australian Journal of Plant Physiology 2001; 28:837–844.

[13] de Morais RF, Quesada DM, Reis VM. Contribution of biological N_2_ fixation to Elephant grass (*Pennisetum purpureum* Schum.). Plant and Soil 2012; 356:23–34.

[14] Eckard NA, Biesboer DD. A survey of nitrogen fixation (acetylene reduction) associated with *Typha* in Minnesota. Canadian Journal of Botany 1988; 66:2419–2423.

[15] Wickstrom CE and Corkran JL. Nitrogenase activities associated with macrophytes from a lacustrine and a freshwater estuarine habitat. Aquatic Botany 1997; 59:157–162

[16] Mwaura FB, Widdowson D. Nitrogenase activity in the papyrus swamps of Lake Naivasha, Kenya Hydrobiologia 1992; 232:23–30.

[17] Prakamhang J, Minamisawa K, Teamtaisong K, Boonkerd N, Teaumroong N. The communities of endophytic diazotrophic bacteria in cultivated rice (*Oryza sativa* L.). Applied Soil Ecology 2009; 42:141–149.

[18] James EK, Baldani JI. The role of biological nitrogen fixation by non-legumes in the sustainable production of food and biofuels. Plant and Soil 2012; 356:1–3.

[19] Keymer DP, Kent AD. Contribution of nitrogen fixation to first year *Miscanthus* x *giganteus*. *GCB* Bioenergy 2014; 6:577–586.

[20] Knoth JL, Soo-Hyung Kim, Ettl GJ, Doty SL. Biological nitrogen fixation and biomass accumulation within poplar clones as a result of inoculations with diazotrophic endophyte consortia. New Phytologist 2014; 201:599–609. doi: 10.1111/nph.12536 24117518 10.1111/nph.12536

[21] Rosenblueth M, Martínez-Romero E. Bacterial endophytes and their interactions with hosts. Molecular Plant-Microbe Interactions 2006; 19:827–837. doi: 10.1094/MPMI-19-0827 16903349 10.1094/MPMI-19-0827

[22] Reinhold-Hurek B, Hurek T. Living inside plants: bacterial endophytes. Current Opinion in Plant Biology 2011; 14:435–443. doi: 10.1016/j.pbi.2011.04.004 21536480 10.1016/j.pbi.2011.04.004

[23] Carpenter HM 2009 Caballitos and Totora: The story of the sedge Schoenoplectus californicus PhD dissertation, University of California, Davis 2009.

[24] Thullen JS, Nelson SM, Cade BS, Sartoris JJ. Macrophyte decomposition in a surface-flow ammonia-dominated constructed wetland: Rates associated with environmental and biotic variables. Ecological Engineering 2008; 32:281–290.

[25] Dejoux C, Iltis A. Lake Titcaca: a synthesis of limnological knowledge Kluwer Academic Publishers, Boston 1992.

[26] Rondon XJ, Banack SA, Diaz-Huamanchumo W. Ethnobotanical investigation of caballitos (*Schoenoplectus californicus*: Cyperaceae) in Huanchaco, Peru. Economic Botany 2003; 57:35–47.

[27] Rejmánková E. Nutrient resorption in wetland macrophytes: comparison across several regions with different nutrient status. New Phytologist 2005; 167:471–482. doi: 10.1111/j.1469-8137.2005.01449.x 15998399 10.1111/j.1469-8137.2005.01449.x

[28] Reich PB, Hungate BA, Luo Y. Carbon-nitrogen interactions in terrestrial ecosystems in response to rising atmospheric carbon dioxide. Annual Review of Ecology, Evolution and Systematics 2006; 37:611–36.

[29] Reed SC, Cleveland CC, Townsend AR. Functional ecology of free-living nitrogen fixation: a contemporary perspective. Annual Review of Ecology, Evolution and Systematics 2011; 42:489–512.

[30] Vitousek PM, Porder S, Houlton BZ, Chadwick OA. Terrestrial phosphorus limitation: mechanisms, implications and nitrogen-phosphorus interactions. Ecological Application 2010; 20:5–15. 10.1890/08-0127.1 20349827

[31] Santi C, Bogusz D, Franche C. Biological nitrogen fixation in non-legume plants. Annals of Botany 2013; 111:743–767. doi: 10.1093/aob/mct048 23478942 10.1093/aob/mct048 PMC3631332

[32] Reed SC, Cleveland CC, Townsend AR. Relationships among phosphorus, molybdenum and free living nitrogen fixation in tropical rain forests: results from observational and experimental analyses. Biogeochemistry 2013; 114:135–147.

[33] DeLuca TH, Zackrisson O, Nilsson M Ch, Sellstedt A. Quantifying nitrogen-fixation in feather moss carpets of boreal forests. Nature 2002; 419:217–220. 10.1038/nature01051 12410308

[34] Boddey RM, de Oliveira OC, Urquiaga S, Reis VM, Olivares FL, Baldani VLD et al Biological nitrogen fixation associated with sugarcane and rice: contributions and prospects for improvement. Plant and Soil 1995; 174:195–209.

[35] Boddey RM, Urquiaga S, Alves BJR, Reis V. Endophytic nitrogen fixation in sugarcane: present knowledge and future applications. Plant and Soil 2003; 252:139–149.

[36] Haiyambo DH, Chimwamurombe PM, Reinhold-Hurek B. Isolation and screening of rhizosphere bacteria from grasses in East Kavango region of Namibia for plant growth promoting characteristics. Current Microbiology 2015; 71:566–571. doi: 10.1007/s00284-015-0886-7 26254764 10.1007/s00284-015-0886-7

[37] Dalton DA, Kramer S, Azios N, Fusaro S, Cahill E, Kennedy Ch. Endophytic nitrogen fixation in dune grasses (*Ammophila arenaria* and *Elymus mollis*) from Oregon. FEMS Microbial Ecology 2004; 49:469–479. 10.1016/j.femsec.2004.04.010 19712295

[38] Terakado-Tonooka J, Fujihara S, Ohwaki Y. Possible contribution of *Bradyrhizobium* on nitrogen fixation in sweet potatoes. Plant and Soil 2013; 367:639–650.

[39] Rout ME, Chrzanowski TH. The invasive *Sorghum halepense* harbors endophytic N_2_-fixing bacteria and alters soil biogeochemistry. Plant and Soil 2009; 315:163–172.

[40] Stal LJ. Nitrogen fixation in cyanobacterial mats. Methods in Enzymology 1988; 167:474–484.

[41] Montoya JP, Voss M, Kähler P, Capone DG. A simple, high-precision, high-sensitivity tracer assay for N_2_ fixation. Applied Environmental Microbiology 1996; 62:986–993 16535283 10.1128/aem.62.3.986-993.1996 PMC1388808

[42] Zehr JP, Montoya JP. Measuring N_2_ fixation in the field In: Bothe H, Ferguson SJ, Newton WE(eds) Biology of nitrogen cycle. Elsevier B.V, Amsterdam, pp 452 2007.

[43] McNamara AE, Hill WR. UV-B irradiance gradient affects photosynthesis and pigments but not food quality of periphyton. Freshwater Biology 2000; 43:649–662.

[44] Phillips DL, Gregg JW. Uncertainty in source partitioning using stable isotopes. Oecologia 2001; 127:171–179; the mixing model can also be found at (accessed 6.1.2017): https://www.google.com/?ion=1&espv=2#q=ISOERROR+1.04+7%2F20%2F2001+Microsoft+Excel+2000+worksheet+to+accompany%3A). doi: 10.1007/s004420000578 24577646 10.1007/s004420000578

[45] Henry S, Baudion E, López-Guitérez JC, Martin-Laurent F, Brauman A, Philippot L. Quantification of denitrifying bacteria in soils by *nir*K gene targeted real-time PCR. Journal of Microbiological Methods 2004; 59:327–335. doi: 10.1016/j.mimet.2004.07.002 15488276 10.1016/j.mimet.2004.07.002

[46] Gaby JC, Buckley DH. A Comprehensive evaluation of PCR primers to amplify the *nif*H gene of nitrogenase. PLOS ONE 2012; 7:e42149 doi: 10.1371/journal.pone.0042149 22848735 10.1371/journal.pone.0042149 PMC3405036

[47] Sokal R R, Rohlf F J. Biometry 3^rd^ Edition Freeman & Comp New York 1995, 887 p.

[48] Asis CA Jr., Shimizu T, Khan MW, Akao S. Organic acid and sugar contents in sugarcane stem apoplast solution and their role as carbon source for endophytic diazotrophs. Soil Science and Plant Nutrition 2003; 49:915–920.

[49] Gyaneshwar P, James EK, Reddy PM, Ladha JK. *Herbaspirillum* colonization increases growth and nitrogen accumulation in aluminium-tolerant rice varieties. New Phytologist 2002; 154:131–145.

[50] Boddey RM, Baldani VLD, Baldani JI, Döbereiner J. Effect of inoculation of *Azospirillum* spp. on the nitrogen assimilation of field grown wheat. Plant and Soil 1986; 95:109–121.

[51] Taulé C, Mareque C, Barlocco C, Hackembruch F, Reis VM, Sicardi M et al The contribution of nitrogen fixation to sugarcane (*Saccharum officinarum* L.), and the identification and characterization of part of the associated diazotrophic bacterial community. Plant and Soil 2012; 356:35–49.

[52] Malik KA, Rakhshanda B, Mehnaz S, Rasul G, Mirza MS, Ali S. Association of nitrogen-fixing, plant-growth-promoting rhizobacteria (PGPR) with kallar grass and rice. Plant and Soil 1997; 194:37–48.

[53] Hedin LO, Brookshire ENJ, Menge DNL, Barron AR. The nitrogen paradox in tropical forest ecosystems. Annual Review of Ecology, Evolution and Systematics 2002; 40:613–35.

[54] Wurzburger N, Hedin LO. Taxonomic identity determines N_2_ fixation by canopy trees across lowland tropical forests. Ecology Letters 2016; 19:62–70. doi: 10.1111/ele.12543 26584690 10.1111/ele.12543

[55] Ritchie ME, Raina R. Effects of herbivores on nitrogen fixation by grass endophytes, legume symbionts and free-living soil surface bacteria in the Serengeti. Pedobiologia 2016; 59: 233–241.

[56] Högberg P. Tansley Review No. 95,^15^N natural abundance in soil-plant systems. New Phytologist 1997; 137:179–203. 10.1046/j.1469-8137.1997.00808.x 33863175

[57] Shearer G, Kohl DH. N_2_-fixation in field settings: estimations based on natural ^15^N abundance. Australian Journal of Plant Physiology 1986; 13:699–756.

[58] Evans RD. Physiological mechanisms influencing plant nitrogen isotope composition. TRENDS in Plant Science 2001; 6:121–126. 11239611 10.1016/s1360-1385(01)01889-1

